# A Daily Accumulation Model for Predicting PFOS Residues in Beef Cattle Muscle After Oral Exposure

**DOI:** 10.3390/toxics13080649

**Published:** 2025-07-31

**Authors:** Ian Edhlund, Lynn Post, Sara Sklenka

**Affiliations:** Division of Food Compliance, Office of Surveillance and Compliance, Center for Veterinary Medicine, United States Food and Drug Administration, College Park, MD 20740, USA; lynn.post@fda.hhs.gov (L.P.); sara.sklenka@fda.hhs.gov (S.S.)

**Keywords:** per- and polyfluoroalkyl substances (PFAS), perfluorooctane sulfonate (PFOS), beef cattle, daily accumulation model (DAM), physiologically based toxicokinetics (PBTK), variable diet

## Abstract

Per- and polyfluoroalkyl substances (PFAS) have been found worldwide in water, soil, plants, and animals, including humans. A primary route of exposure for humans and animals to PFAS is through the diet and drinking water. Perfluorooctane sulfonate (PFOS), a long-chain PFAS with a relatively long half-life, has been associated with adverse health effects in humans and laboratory animals. There are few toxicokinetic studies on PFOS in domestic livestock raised for human food consumption, which are critical for assessing human food safety. This work aimed to develop a simple daily accumulation model (DAM) for predicting PFOS residues in edible beef cattle muscle. A one-compartment toxicokinetic model in a spreadsheet format was developed using simple calculations to account for daily PFAS into and out of the animal. The DAM was used to simulate two case studies to predict resultant PFOS residues in edible beef cattle tissues. The results demonstrated that the model can reasonably predict PFOS concentrations in beef cattle muscle in a real-world scenario. The DAM was then used to simulate dietary PFOS exposure in beef cattle throughout a typical lifespan in order to derive a generic bioaccumulation factor. The DAM is expected to work well for other PFAS in beef cattle, PFAS in other livestock species raised for meat, and other chemical contaminants with relatively long half-lives.

## 1. Introduction

The worldwide distribution of per- and poly-fluoroalkyl substances (PFAS), their persistence in the environment, toxicological studies in laboratory animals, and epidemiological studies in humans has created a flurry of public, scientific, and regulatory activity to determine human health risks. PFAS comprise more than 4000 chemicals to include two classes of fully fluorinated perfluoroalkyl acids (PFAA), perfluorooctanoate (PFOA), and perfluorooctane sulfonate (PFOS) [1,2,3]. PFAA compounds are amphiphilic and anionic (hydrophilic heads and hydrophobic tails) and widely used as surfactants in industrial and consumer products, such as fire retardants, aqueous firefighting foam, food packaging materials, and non-stick cookware, due to their temperature and chemical stability. These same stability properties make them resistant to degradation by metabolism, hydrolysis, sunlight, and biodegradation, which allows for bioaccumulation and indefinite persistence in the environment.

Increasing costs of commercial fertilizers and sewage sludge disposal have driven the use of biosolids to improve soil fertility [4]. PFAA are present in aqueous effluents and treated biosolids from wastewater treatment plants [5]. The application of biosolids as soil amendments on crop land may result in bioaccumulation of PFAA in grain crops and produce, such as lettuce and tomatoes [6]. PFOA and PFOS concentrations in biosolids range from 1 to 244 μg/kg of dry matter (DM) and from 5 to 3120 μg/kg DM, respectively, in the United States [7]. The sum of 24 PFAS concentrations in biosolids ranged from 1 to 3200 μg/kg and averaged 108 ± 277 μg/kg DM, where PFAS with carboxyl and sulfonic functional groups comprised 29% and 71% of the total 24 PFAS concentrations, respectively, on average [8]. Crops grown in soils amended with biosolids have been found to contain PFOA and PFOS concentrations of 10–200 μg/kg DM and 1–20 μg/kg DM, respectively, from tall fescue, barley, Bermuda grass, and Kentucky bluegrass [9]. Well and ground water are other routes of human PFAS exposure where biosolids have contaminated these water sources [10,11,12].

PFAS have been found worldwide in drinking water and the human diet, including in meat, milk, eggs, and seafood, and serve as sources of PFAS exposure in humans [13,14,15,16,17,18,19,20,21,22]. Human epidemiological studies have associated PFAS chemicals, particularly PFOA and PFOS, to altered immune and thyroid function, kidney and liver disease, lipid and insulin dysregulation, adverse reproductive and fetal development outcomes, and cancer [23]. Many of the human adverse effects of PFOS have been observed in laboratory animals, such as weight loss, reduced serum cholesterol and thyroid hormones, and hepatoxicity [24]. Mice, rats, and rabbits exposed to PFOS had reduced fetal weight, cleft palate, and reduced neonatal survival [25]. For PFOA, induction of liver tumors, Leydig cell tumors, and pancreatic acinar cell tumors were observed in rodents along with increased post-weaning mortality, decreased body weight (*BW*), and delayed puberty [26,27,28].

Long-chain PFAA greater than 7 fluorinated carbons are proteinophilic (hydrophobic and lipophobic) and distributed mainly to serum albumin, liver, and kidneys in rats [24,29]. PFOS, perfluorohexane sulfonic acid (PFHxS), and PFOA were 99% bound to serum albumin of rats, bovines, or humans [30,31,32]. The liver concentrations are 3–5 times greater than the serum concentration in rats but only about 1.3–2 times greater in humans and non-human primates [24]. PFOA plasma half-lives differ across female (24 h.) and male (105 h.) rats but not for PFOS with a half-life of 30–50 days for rats and 40 days for mice [33,34,35]. There are large differences across species, where monkey half-lives are 21–30 days for PFOA and 4–6 months for PFOS compared to human half-lives of 3–5 years for both PFAAs [30,36,37,38]. The elimination half-lives in chickens raised for meat and eggs are 3.9–4.6 days for PFOA and 17–125 days for PFOS compared to the longer half-lives of 236 days and 634 days for pigs, respectively [39,40,41,42]. For cattle, the half-life for PFOA of 19.2 h to 1.3 days is relatively short compared to PFOS elimination with half-lives of 38.7–165 days depending on the experimental design [43,44,45,46,47]. Like beef cattle, different toxicokinetics (absorption, distribution, and excretion) were observed in dairy cattle, where plasma concentrations were <2 μg/L for PFOA and >2250 μg/L for PFOS during the 29-day depuration period [48]. A pilot study with 2 sheep also showed a large toxicokinetic difference in median plasma concentrations of PFOA (2.7 μg/L and 16.4 μg/L) and PFOS (32.1 μg/L and 50.3 μg/L), where steady state was not achieved before the 21-day depuration period [49].

There are relatively few toxicokinetic studies for PFOS and PFOA in tissues of domestic animals raised for human food consumption compared to the many studies for ground water [50]. Toxicokinetic studies in chickens have provided some information for eggs and broiler meat, and pork from one swine study [38,40,41,42,51]. For ruminants, there were single bolus dosing studies in beef cattle and short-term feeding studies in dairy cattle and sheep consuming contaminated forage [43,45,46,48,49,52,53]. A long-term study of about 9 months duration was conducted for dairy cattle consuming background concentrations of PFOS and PFOA from forage (<7 ng/kg and <14 ng/kg, respectively) and drinking water (<0.08 ng/L and <0.24 ng/L, respectively) [44]. Another long-term study explored plasma and skin concentrations of PFOS, PFOA, PFHxS, and perfluorooctane sulfonic acid (PFHpS), as the major contaminants, using 180 dairy cattle from a herd of 5000 animals after life-time exposure to contaminated feed and water [54].

The previous toxicokinetic studies in food animals provide a foundation of useful toxicokinetic parameters, such as half-life, but fall short of a modeling approach that includes fluctuations of water and dry matter intake (DMI) over time to predict serum and tissue concentrations of PFAS [55]. Differences in PFAS intake that are affected by fluctuations in water and DMI over time (seasons) are unlikely to reach a steady state. For PFOS, steady state may be achievable after consistent intake for >4 half-lives of 38.7–120 days or about 5–16 months for cattle [43,44,45,46,47,48,55,56]. Consequently, steady state toxicokinetics for PFOS are not a reliable estimate of serum and tissue concentrations because of large differences between actual measurements (experimental) and computational estimates (predicted) [55]. Daily exposures to PFOS and PFHxS were estimated for water, soil, and feed intakes that included allowance for growth of the grazing cattle, seasonal variation, and variable PFAS concentration across pastures [57]. The daily exposure intakes were used to develop a simple, validated, and comprehensive toxicokinetic model that incorporated variations of water, soil, and DMI for a population of beef cattle over time. An interactive generic physiologically based toxicokinetic (PBTK) study for beef and dairy cattle estimated distribution and depuration rates for PFOA, PFOS, and PFHxS in liver, kidney, muscle, and the rest of the animal for beef cattle and the same model for dairy cattle but including udder and milk compartments [58]. Since there were no protein binding, absorption or elimination rate constants, partition coefficients, enterohepatic circulation, or renal reabsorption data for cattle, the values used were from previously published PBTK models for PFOA and PFOS in rats [59,60]. Simulations of the generic PBTK models for beef and dairy cattle were validated against independent data sets [61,62]. However, actual exposure scenarios were not available for PFAS concentrations in dietary sources, but were based on measurements of PFOA and PFOS concentrations in water and soil samples [63,64].

The purpose of this work is to present a simple, comprehensive PFOS daily accumulation model (DAM) for beef cattle from birth to slaughter that is independent of steady state toxicokinetics. Although a more complex PBTK is a more precise approach, some crucial parameters for cattle are not available in the scientific literature. Likewise, a model for PFAS mixtures would be ideal, but only one PFAS (PFOS) is considered in the DAM until there is a viable approach for modeling PFAS mixtures. Also, the DAM may be suitable for other PFAS, such as PFHxS, if properly parameterized. Thus, the DAM is a hybrid model combining elements of PBTK, such as depletion toxicokinetics, with exposure scenarios from birth to slaughter to calculate daily PFOS intakes for water, milk, soil, and forage.

## 2. Materials and Methods

The DAM is a one-compartment toxicokinetic model in a spreadsheet format with a separate row for each day in an animal’s life. This format allows for maximum flexibility in parameterization, such as exposure and *BW*, while also being straightforward to use for the average risk assessor with limited computer programming experience.

The model relies on simple PFAS in and out calculations. The mass of PFAS into the animal is the amount of PFAS in the diet, including soil, water, and food. Additional routes of exposure, such as inhalation or dermal, could be added if warranted by a specific use case. The DAM relies on the assumption of 100% PFAS absorption in the gut, which is common among other PFAS livestock models [50] but could be modified if that assumption is found to be inaccurate for a specific use case. For the cases included in the present study, the DAM begins with no PFOS in the calf, a value that could easily be changed if the modeler were to calculate fetal PFOS transfer from cow to calf. The mass of PFAS out of the animal is calculated using a half-life equation (Equation (1)). The mass of PFAS in the animal is kept as a running log, adding the PFAS in and subtracting the PFAS out each day.

(1) $$Daily\;elimination\;\left(\mu g\right)=Amt\;in\;body\;(\mu g)-(Amt\;in\;body\;(\mu g)\times {0.5\;}^{\frac{1}{half-life\;(days)}})$$

The concentration of PFAS in the muscle is calculated by multiplying the concentration of PFAS in the entire body by the muscle:body PFAS ratio.

### 2.1. Parameters

Parameters utilized in the DAM were gathered from the literature. Wherever possible, a weight of evidence (WOE) approach was used to calculate a best value and range of possibilities for each parameter. The range of each parameter is necessary for probabilistic (Monte Carlo) modeling.

#### 2.1.1. PFOS Half-Life

**Figure 1:**
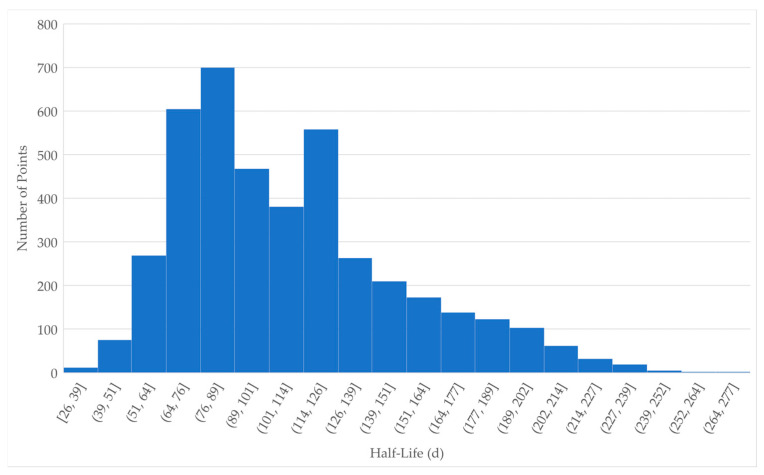
Distribution of half-life values for PFOS. Individual values were randomly selected from each of the distributions defined in Table 1. The number of values from each distribution were according to the Total Weight of each study.

PFOS half-life in beef cattle has been calculated for various tissues from in vivo case studies [46,47,52]. One study concluded that PFOS half-lives in beef cattle vary among different tissues [46], while another study concluded that the half-lives in different tissues are essentially the same [47]. Half-life values from each of these studies were weighted as applicable to the specific use cases presented in this study: long-term, daily dose, and dietary exposure with residues measured in the blood and muscle. The weights used (shown in Table 1) are for the type of exposure (1 for single, 3 for daily), ingestion (1 for water, 3 for food), tissue sampled (1 for blood, 3 for muscle or whole body), and duration of experiment (1 for acute, 3 for chronic). Study divisors were applied for studies that provided values in multiple tissues, to limit the contribution of a single study. The weights for each category were applied to give a total weight for each value shown in Table 1. A combined distribution was formed by randomly selecting 100× the total weight points from within the distribution of each value, resulting in a set of 4200 points (Supplementary Worksheet SW1) for the combined distribution shown in Figure 1. Standard deviations (SD) for PFOS in heifer plasma [46] and for PFOS in serum [47] were approximately 21.8% and 18.1% of the respective mean values; where a SD was not available for a given value, 20 percent of the value was used as a default. The resulting half-life parameter for PFOS in beef cattle used in the model is 108.9 ± 40.5 days.

#### 2.1.2. Muscle:Body PFOS Ratio

The *Muscle:Body PFOS ratio* is a decimal number that is used to determine the mass of PFOS present in the muscle as compared to the entire body. The calculation is based on the assumption that PFOS is rapidly distributed through tissues relative to the duration of the study or the half-life of PFOS. In circumstances where there is a drastic shift in exposure, the predicted PFOS concentrations in some tissues over the next few days of the simulation may be inaccurate as the PFOS finds its new equilibrium. The ratio was calculated using the mass balance tissue concentrations from two in vivo studies [46,47]. The studies were long term (>30 days) and provided tissue concentrations in several of the primary PFOS tissue reservoirs, including, at least: blood, liver, kidney, and muscle. One study also included data for bone and fat. For determination of mass balance, we assumed that all PFOS in the body was contained in the measured tissues. Equal weighting was applied to both studies and the average value for steers and heifers was used. The SD was calculated between the values of the two studies. The resulting *Muscle:Body PFOS ratio* in beef cattle was 0.231 ± 0.090 (unitless). A similarly calculated muscle:serum ratio was calculated and is used to predict the PFOS concentration in the serum. A distribution for the muscle:serum ratio was not available since only one value was available in the literature; therefore, a default 20 percent value was used for probabilistic simulation. Further details of partitioning values and calculations are included in the Supplementary Worksheet SW2.

We can predict the *Mass of PFOS in Muscle* using Equation (2):

(2) Mass of PFOS in muscle=Muscle:Body PFOS Ratio×Mass of PFOS in body

The *Concentration of PFOS in Muscle* can then be calculated using the *BW* and the *Muscle BW Fraction*, as in Equation (3). A value of 0.361 ± 0.117 kg muscle/kg *BW* for the *Muscle BW Fraction* was found in the literature for relative organ weights of beef cattle [65]; calculations are included in Supplementary Worksheet SW2.

(3) $$Concentration\;of\;PFOS\;in\;muscle=\frac{Mass\;PFOS\;in\;muscle}{Muscle\;BW\;fraction\times BW}$$

#### 2.1.3. Weight and Growth

A growth equation for Holstein beef steers [65] was used to derive daily *BW* and is shown in Equation (4), where “*d*” is the age in days. Growth is breed dependent, and while Angus is the predominant beef cattle breed in the United States, variability was factored into the growth parameter to account for inter- and intra-breed differences in growth.

(4) $$BW\;\left(kg\right)=35.45+0.5584d+{0.001787d}^{2}-{1.549\times 10^{-6}d}^{3}-1.332\times 10^{-9}d^{4}$$

To account for variation in body weight, the DAM uses a daily weight gain added to either the birth weight (for day 0) or the previous day’s weight for the following days. Rather than using the birth weight for Holsteins [65], an average birth weight among all breeds of cattle (35.45 ± 3.27 kg) was used [66]. Taking the derivative of Equation (4) gives the rate of change of weight (the daily weight gain) of the animal, shown in Equation (5).

(5) $${BW}^{\prime }=0.5584+2\times 0.001787d-{3\times 1.549\times 10^{-6}d}^{2}-4\times 1.332\times 10^{-9}d^{3}$$

The weight gain equation is used until an age of 540 days, after which, a consistent gain of 0.32 kg/day is assumed. Using an initial birth weight (and range) and a daily weight gain (with variability) provides a practical range of *BWs* to be used in probabilistic modeling.

#### 2.1.4. Forage and Milk Intake for Nursing Calves

**Figure 2:**
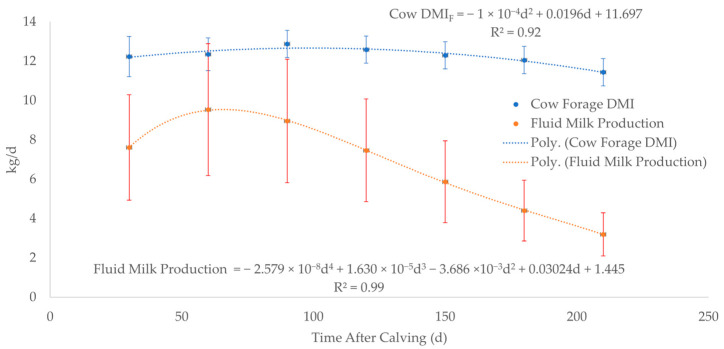
Cow forage dry matter intake (*DMI_F_*) and fluid milk production at 30-day intervals from days 0 to 210. The dashed lines represent the best fit equation (predicted) of the data points (solid dots) with the associated error bars of the actual data.

**Figure 3:**
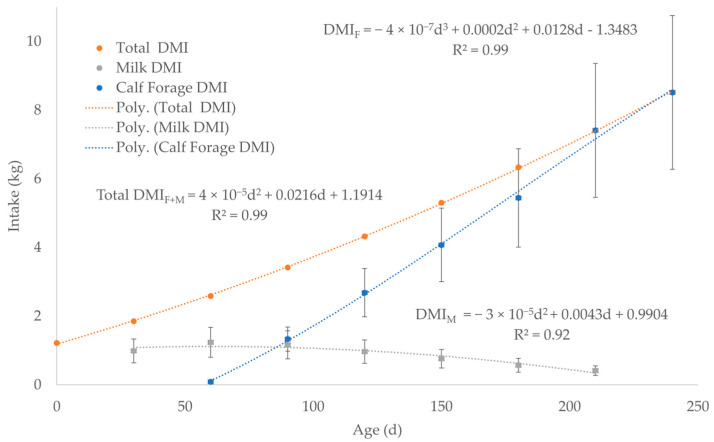
Forage dry matter intake (*DMI_F_*) for nursing calves was calculated by subtracting milk dry matter intake (*DMI_M_*) from total milk plus forage dry matter intake (DMI_F+M_) before weaning at 240 days of age. The dashed lines represent the best fit equation (predicted) of the data points (solid dots) with the associated error bars of the actual data.

Forage dry matter intake (*DMI_F_*) for cows (Figure 2, Supplementary Worksheet SW3) was estimated by averaging the *DMI_F_* for 3 levels of nutrition during 7 months of lactation since calving from the data in the National Research Council (NRC), 2000 Nutrient Requirements for Beef Cattle [67]. Fluid milk production (Figure 2, Supplementary Worksheet SW3) was estimated by averaging 4 lactation curves for peak production at 5, 8, 11, and 14 kg/day using the chart in the NRC, 2000 Nutrient Requirements for Beef Cattle [67]. WebPlotDigitizer was used to extract numerical data from each of the 4 lactation curves at 30-day intervals from birth to 210 days of age (Supplementary Worksheet SW4). Forage DMI for nursing calves was calculated by subtracting milk DMI (*DMI_M_*) from total forage plus milk DMI (DMI_F+M_) up to weaning at 240 days of age (Equation (6), Figure 3, Supplementary Worksheet SW4) [68]. The average forage digestible energy (*DE_F_*) of 2.27 Mcal/kg in Equation (6) was calculated using the average of 50 forages compiled from a table in NRC, 2007 Nutrient Requirements of Horses (Supplementary Worksheet SW4) [69].

The investigators experimentally validated Equation (6) from 50 to 200 kg *BW*, which approximates 25 to 206 days of age by using Equation (4) to back calculate from *BW* to age [68]. In Equation (6), there is little need to adjust for *DMI_F_* increase due to *DE_F_* increase, because the *DMI_M_* from 30 to 210 days of age in Figure 3 essentially overlaps 25 to 206 days of age (50 to 200 kg *BW*). Another limitation of Equation (6) is that it does not account for forage quality because Equation (6) was developed by the investigators for a single forage, alfalfa hay, with an average *DE_F_* of 3.18 Mcal/kg [68]. However, as *DMI_F_* and *BW* increases, total DE from forage increases for animals with a functional rumen associated with an increased energy demand for growth because ruminal fermentation increases the ability of the calf to consume fibrous forages [68,69]. Although alfalfa hay is a good quality forage to meet the increased energy demand, pasture calves may not have access to high quality forage. Thus, the average *DE_F_* value of 2.27 Mcal/kg for 50 forages accounts for some variation in forage quality (Figure 3, Supplementary Worksheet SW4), which conservatively increases the *DMI_F_* compared to a high-quality forage, such as alfalfa with a *DE_F_* of 3.18 Mcal/kg.

(6) $${Calf\;DMI}_{F}\;(kg)=\left\lceil \left(DEI\times BW\right)-\left({DE}_{M}\times {DMI}_{M}\right)\right\rceil /{DE}_{F}$$

where,

*Calf DMI_F_* is the calf dry matter intake of forage (kg).

*BW* is calculated using Equation (4) at 30-day intervals from birth to weaning at 240 days of age.

Mcal is megacalories (1000 calories).

*DEI* is the average daily energy intake of forage plus milk estimated at 0.0783 Mcal/kg BW per day over a 200-day period of the nursing calf [68].

*DMI_M_* is the dry matter intake of milk. If the nursing calf consumes 100% of the fluid milk production (Figure 2) and fluid milk is approximately 13% dry matter, DMI_M_ is calculated at 30-day intervals from birth to 210 days of age.

*DE_M_* is the digestible energy of milk estimated at 4.87 Mcal/kg of DMI_M_ [68].

*DE_F_* is the average forage digestible energy (2.27 Mcal/kg) calculated from 50 forages [69].

#### 2.1.5. Forage Intake from Birth to Slaughter

**Figure 4:**
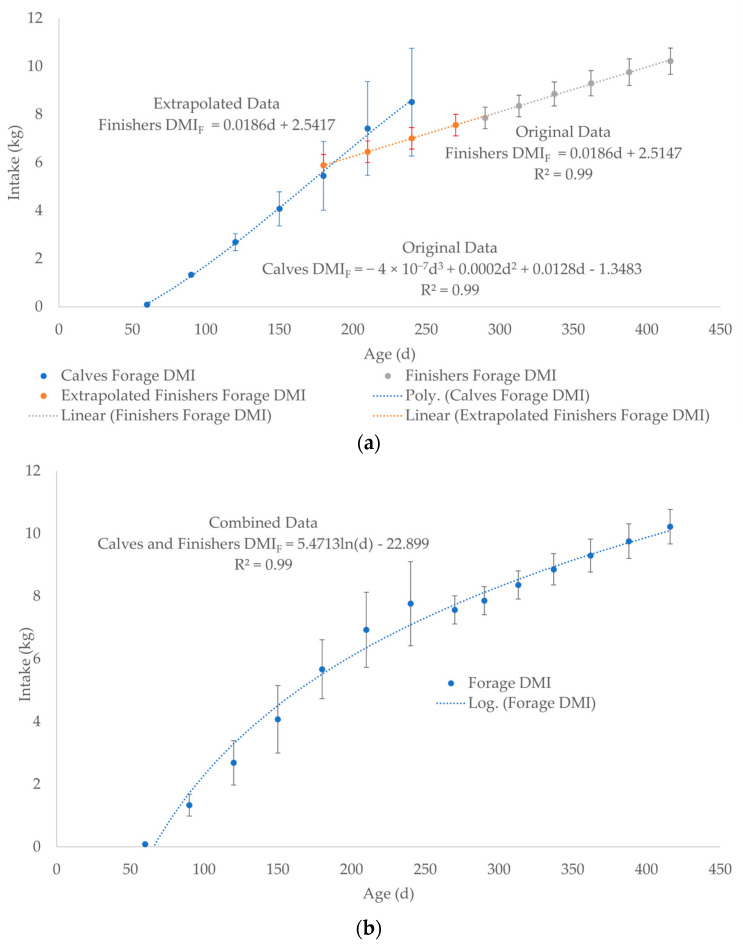
(**a**) Separate data plots as two equations: *DMI_F_* for calves (polynomial trendline) and finishers (linear trendline) as Original Data. The linear trendline for the finishers *DMI_F_* was back extrapolated using to 180 days of age using the equation: *DMI_F_* = 0.0186d + 2.5147 in Figure 4a (Extrapolated Data). (**b**) The data for calves and finishers were combined (Combined Data) into a single logarithmic trendline and equation for *DMI_F_* from birth to slaughter. The Original Data at 180, 210, and 240 days of age for calves in Figure 4a was averaged with the Extrapolated Data for finishers. The dashed lines represent the best fit equation (predicted) of the data points (solid dots) with the associated error bars of the actual data.

*DMI_F_* for calves after weaning (finishers) was estimated by averaging the DMI for 5 levels of nutrition from 300 to 425 kg *BW* at 25 kg increments of growth (Original Data, Figure 4a, Supplementary Worksheet SW5) using data in the NRC, 2000 Nutrient Requirements for Beef Cattle [67]. The 6 *BW* increments were converted to days of age by back calculating from *BW* using Equation (4) to generate a linear *DMI_F_* after weaning (Figure 4a, Supplementary Worksheet SW5). Then, the linear trendline was back extrapolated to 180 days using the equation from Figure 4a: *DMI_F_* = 0.0186d + 2.5147 (Extrapolated Data, Figure 4a, Supplementary Worksheet SW5). The *DMI_F_* data for calves (Original Data) were averaged with Extrapolated Data for finishers at 180, 210, and 240 days of age (Figure 3 and Figure 4a, Supplementary Worksheet SW4 and SW5). The data point of 7.56 kg of *DMI_F_* at 270 days of age (279 kg *BW*) was also calculated using the equation in Figure 4a: *DMI_F_* = 0.0186d + 2.5147 (Supplementary Worksheet SW5). The Original and Extrapolated Data sets were combined in one data set (Combined Data) to generate a single logarithmic curve for the *DMI_F_* of calves and finishers from birth to slaughter (Figure 4b, Supplementary Worksheet SW5). The Combined Data set was an effort to account for the uncertainty of not only the age at weaning but also variations in forage and milk DMI from 180 to 240 days of age (Figure 4b, Supplementary Worksheet SW5).

#### 2.1.6. Soil and Water Intake

The average water intake for lactating cows and finishers was calculated using data from NRC, 2000 Nutrient Requirements of Beef [67]. For lactating cows, the average water intake of 56.47 L/day was calculated from 4.4 to 32.2 °C at increments of 4.4 °C (Supplementary Worksheet SW6). For finishers, the average water intake was calculated across 4.4–32.2 °C at increments of 4.4 °C for each 273, 364, and 454 kg *BW* category; then, the three resulting water intakes were averaged to yield a final value of 40.84 L/day (Supplementary Worksheet SW6). The water intake for calves was calculated using Equation (7). Soil intake for cows, calves, and finishers grazing on pasture was estimated at 2.43% of *DMI_F_* [70].

(7) $${WI}_{calf}\;\left(\frac{L}{day}\right)=4\times {DMI}_{F}$$

where,

*WI_calf_* is the water intake of the nursing calf (L/day).

*DMI_F_* is forage dry matter intake (kg/day) and does not include the water from fluid milk intake [71].

Four (4) is the rate of water intake per kg of *DMI_F_* (L/kg/day).

### 2.2. Monte Carlo Analysis

Monte Carlo analyses of the simulations were conducted using the @RISK package for Excel by running 1000 iterations with parameter values randomly selected from within their respective distributions. Distributions for each of the model parameters were discussed above and are shown in Table 2 and Supplementary Worksheet SW7. The distributions were truncated at the fifth and ninety-fifth percentiles to reduce mathematical issues with calculated fields.

### 2.3. Case Studies

A single study site served as the basis for two in vivo case studies. The site was a small beef farm that raised Angus cattle and grew forage onsite for its animals. The cattle received farm-grown forage for half the year during the winter months and grazed on pasture in the summer. Fields used to grow forage, including dry hay and hay silage, had a long history of biosolid application while pasture fields used for grazing did not. One hay field, however, was also used for cattle grazing for approximately 6 weeks each year after the hay was harvested. Thus, the cattle had varied exposure to PFOS depending on the time of year and where their food was sourced. While animals had access at times to pasture surface water, their preferred and primary water source came from the farm’s groundwater well.

Environmental samples including field soils and some collocated forage plants, pasture soils, fresh hay cuttings, ground well water, and surface water were collected. Details regarding sample collection procedures, as well as additional details on the study environment and practices, can be found in Astmann et al. (2025) [72]. Specific feeding history for this case study, which was used to parameterize the DAM, is shown in Table S17 of the study [72].

Each of the in vivo data sets had different data available regarding what was fed to the animals and when. Whenever possible, actual data collected during the case studies (such as PFOS concentrations in the forage and water for times when animals were exposed, approximate ages, etc.) were input into the DAM. Where data were unavailable, data (such as daily BW, forage/water/milk intake, etc.) were assumed based on standard practices for raising beef cattle, as described above.

## 3. Results

The DAM was used to simulate two different in vivo case studies of beef cattle grown for slaughter to predict resultant PFOS residues in beef muscle tissue. In the second case, a prediction was also made for serum PFOS concentrations.

### 3.1. Case Study 1

For Case Study 1, frozen beef muscle tissue from a two-year old animal raised on the study site was collected and analyzed for PFOS. The concentration of PFOS found in this beef muscle tissue sample was identified as shown in Table S11 of Astmann et al. (2025) and used to validate the parameterized DAM [72].

**Figure 5:**
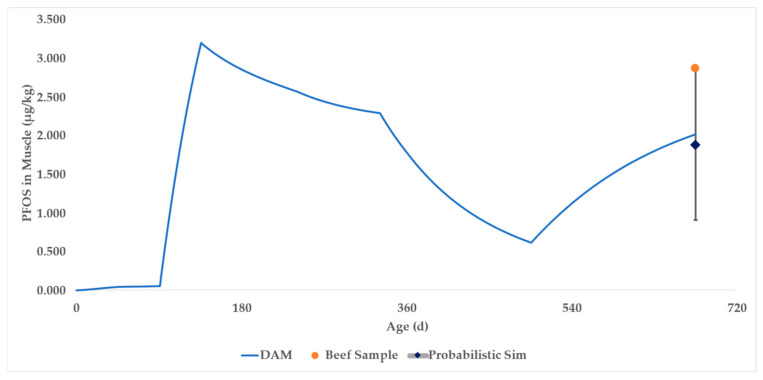
Case Study 1 simulation results for PFOS in beef muscle. The blue curve represents the output of the deterministic DAM. The dark blue diamond represents the mean of the probabilistic DAM, with associated error bars representing the 90% confidence interval. The orange dot represents actual sampling data.

The DAM was parameterized and simulated for 675 days (Supplementary Worksheet SW8) to correspond with the life of the beef steer. The PFOS muscle concentration in the final row of the deterministic model was 2.02 μg/kg PFOS, compared to the average in vivo result of 2.87 μg/kg PFOS (shown as an orange point in Figure 5 and SW9). The blue curve represents the DAM time course prediction of PFOS in beef muscle.

A probabilistic simulation was performed (Supplementary Worksheet SW10), resulting in a mean of 1.89 ± 0.59 (SD) μg/kg PFOS (shown as a dark blue diamond in Figure 5). The 90% confidence interval (CI) of the simulations encompasses 0.91 to 2.87 μg/kg PFOS and is shown as error bars above and below the dark blue diamond in Figure 5.

### 3.2. Case Study 2

For Case Study 2, a depuration study was conducted using beef cattle from the study site. Five animals were provided PFAS-free feed for several months prior to being slaughtered. PFAS levels were monitored through collection and analysis of muscle tissue and serum samples at various timepoints throughout the depuration period. An initial muscle biopsy was taken from all 5 animals at approximately 655 days of age after 2 months of consuming uncontaminated feed. At approximately 735 days of age, a second muscle tissue sample was obtained from the animals at slaughter. Serum samples were taken from each animal at approximately 655, 666, 693, and 735 days of age. Details regarding biological sample collection procedures and subsequent laboratory analysis can be found in Astmann et al. (2025) [72].

The DAM was parameterized with the feeding history from the on-farm sampling as described in Table S18 of the Astmann et al. (2025) study [72]. Between months 17.5 and 20, the 1st and 2nd cut hay feeding scheme was parameterized in accordance with the description immediately preceding Table S2 [72].

**Figure 6:**
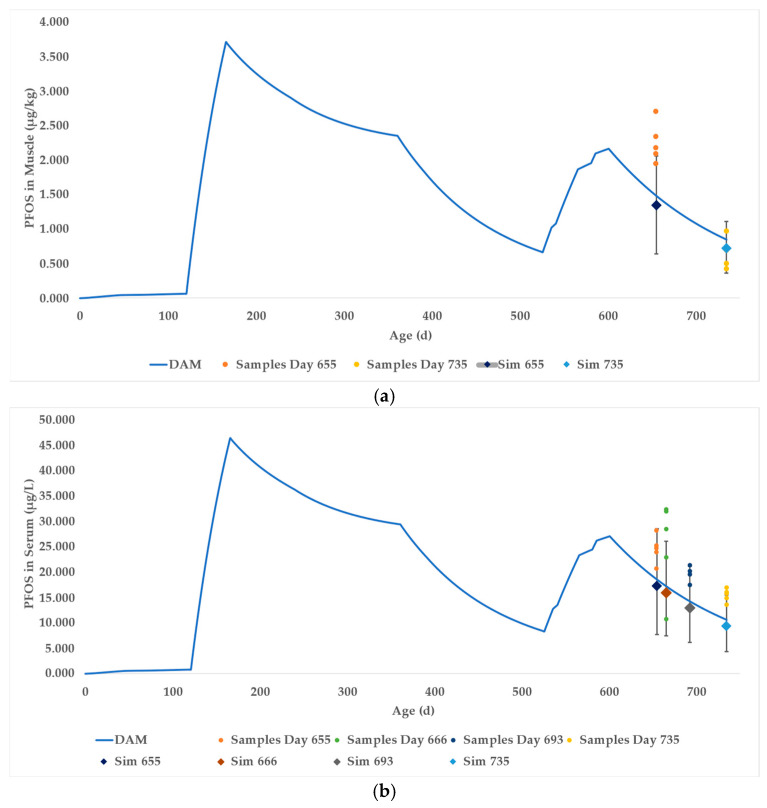
Case Study 2 simulation results of PFOS in (**a**) muscle and (**b**) serum. The blue curves represent the outputs of the deterministic DAMs. The diamonds represent the mean of the probabilistic DAMs at respective time points, with associated error bars representing the 90% confidence intervals. The dots represent actual sampling data.

The DAM was simulated for 735 days (Supplementary Worksheet SW12) to correspond with the lifetimes of the beef cattle. The PFOS muscle concentration of the deterministic model at day 655 was 1.49 μg/kg and at day 735 was 0.85 μg/kg PFOS, approximately 66 percent and 123 percent of the mean in vivo values at day 655 and at slaughter, respectively. The in vivo values for the individual cattle are shown as orange and yellow dots in Figure 6a (and Supplementary Worksheet SW13). The blue curve represents the deterministic DAM time course prediction of PFOS in beef muscle.

A probabilistic simulation (Supplementary Worksheet SW14) was performed by running 1000 Monte Carlo simulations using the distributions described above, resulting in a mean of 1.35 ± 0.42 (SD) μg/kg PFOS at day 655 and 0.73 ± 0.23 μg/kg PFOS in the muscle at slaughter (shown as blue diamonds in Figure 6a). The 90% CI of the simulations encompasses 0.64 to 2.06 μg/kg PFOS at day 655 and 0.36 to 1.11 μg/kg PFOS at slaughter. The mean and range of predictions for the muscle concentrations at day 655 were low, likely due to the DAM not accounting for the slow withdrawal of PFOS in muscle following the sharp change in PFOS exposure preceding the sample collection. The study also did not account for PFOS precursors in the exposure media, which would contribute to a greater than predicted concentration of PFOS in the in vivo data. The 90% confidence interval of predicted PFOS in muscle fully encompasses the in vivo samples at day 735.

Case Study 2 also provided data for serum and plasma PFOS concentration at days 655, 666, 693, and slaughter. Column W in the DAM (Supplementary Worksheet SW12) shows the calculated concentrations of PFOS in the serum. The serum data are shown in Figure 6b using the same scheme as discussed for Figure 6a. The probabilistic DAM simulation distribution encompassed the majority of the in vivo samples, with the notable exception of day 666 when 3 of the in vivo sample PFOS concentrations were inexplicably high.

## 4. Discussion

The deterministic DAM values predicted the mean measured in vivo case study values within thirty-five percent for all measured tissues (blood and muscle) and time points. The probabilistic DAM encompassed the majority of measured values as noted above. These results demonstrate that the DAM can reasonably predict in vivo PFOS concentrations in beef cattle muscle and blood with adequate feeding data. Sudden changes in concentrations of PFOS in the food may result in acute variations of tissue partitioning not accounted for by the DAM. Likewise, exposure to PFOS precursors or other unaccounted for sources of PFOS would result in predicted residues lower than in the in vivo results.

A sensitivity analysis was performed on the probabilistic simulations using @RISK 8.9.0 software. The *Muscle:Body PFOS* fraction was the most sensitive parameter for predicting PFOS in the muscle and blood. The PFOS *Muscle:Serum Fraction* was the second most sensitive parameter for predicting the PFOS concentration in the serum.

*Half-life* and *BW* gain were also sensitive parameters, but since @RISK 8.9.0 rates the contribution for each row separately (for example, the half-life for day 107), the full contribution of these parameters is understated. Likewise, PFOS intake is calculated on a daily basis and, therefore, was understated as well. The sum of the daily contributions of each parameter could be calculated and compared, but that is outside the capability of @RISK 8.9.0. The @RISK 8.9.0 reports, including the top inputs ranked by contribution to variability, are included in the Supplementary Worksheet SW11 and SW15 through SW20.

### Generic Model

The DAM was parameterized for beef cattle on a diet with consistent PFOS concentration throughout an 18-month lifespan to predict a generic bioaccumulation factor (BAF) for beef cattle with chronic exposure to PFOS.

The PFOS concentration in the cattle food was set so that the resulting PFOS concentration in muscle after 540 days would equal the U.S. Department of Agriculture’s (USDA) current minimum level of applicability (MLA) for screening and confirmation in bovine muscle tissue, which is 0.5 μg/kg PFOS [73]. In the generic model, the entire PFOS exposure is from food, so water and soil PFOS concentrations are set to zero.

**Figure 7:**
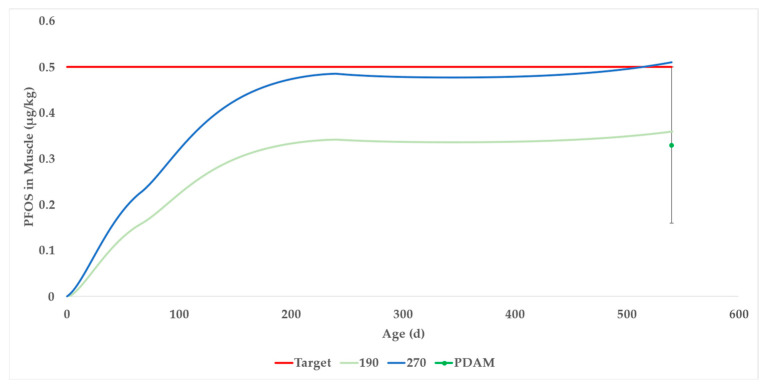
Generic beef DAM was generated to a result of 0.5 μg/kg for PFOS in muscle at slaughter (540 days) by setting a constant 0.270 μg/kg of ration for the deterministic model. For a 95% confidence interval, the PFOS concentration in beef muscle would not exceed 0.5 μg/kg at 540 days by setting a constant of 0.190 μg/kg of ration for the probabilistic model.

The deterministic simulation (Supplementary Worksheet SW21), set to 0.270 μg/kg PFOS constant exposure in the total ration of beef cattle, results in a concentration of 0.501 μg/kg PFOS in beef muscle after 540 days (the blue curve in Figure 7). This number is the best prediction for average beef cattle.

We learned from the data in Case Studies 1 and 2 that within-field variation of PFOS concentrations in forage was approximately forty percent. For a realistic estimate of the PFOS concentrations in the muscle tissues of a cattle herd, this variability was incorporated into the Monte Carlo simulation. In other words, we expect that the PFOS concentration in the forage, even if grown in the same field, would fluctuate and therefore that variation should be incorporated into the simulation. To be 95% confident that the PFOS concentration in beef muscle would not exceed 0.5 μg/kg after 540 days, a modeler should refer to Supplementary Worksheet SW22–SW24 (the green dot in Figure 7 with error bars at slaughter). In that case, 0.190 μg/kg PFOS in the total ration results in a 95th percentile PFOS concentration in beef muscle of 0.504 μg/kg at slaughter.

Dividing a PFOS muscle concentration of 0.501 μg/kg by the total ration PFOS concentration of 0.270 μg/kg gives a deterministic (simulated) BAF of 1.86. The 95th percentile of the probabilistic model results in a BAF of 2.64.

## 5. Conclusions

The DAM represents a novel approach in the field of PFAS toxicokinetics in livestock, bridging the gap between overly simplistic steady-state models and complex PBTK models. As a one-compartment toxicokinetic model in spreadsheet format, it provides flexibility in parameterization and is user-friendly for risk assessors with limited programming experience. Unlike previous models, such as the interactive generic PBTK model for beef and dairy cattle [58], the DAM offers a more accessible tool while still accounting for crucial factors, like variable diet and growth patterns. Compared to the dynamic exposure model [57], the DAM provides a more straightforward implementation while maintaining the ability to predict PFOS concentrations throughout an animal’s lifespan.

The DAM’s key advantage lies in its independence from steady-state toxicokinetics, allowing it to account for fluctuations in water and food intake over time. The deterministic model demonstrated reasonable predictive capabilities, close to in vivo case study values, and the probabilistic DAM encompassed the majority of measured values. As George Box said, “All models are wrong, but some are useful.” The DAM embodies this philosophy, offering a practical trade-off between complexity and utility.

However, the model has several limitations that should be considered. As a simplified model, it may not capture the full complexity of PFOS distribution in different tissues. The assumption of 100% PFAS absorption in the gut may not always be accurate, and other exposure sources, such as inhalation and dermal, are not accounted for. The model is currently limited to PFOS, not accounting for PFAS mixtures or other individual PFAS compounds. The rapid distribution assumption may lead to inaccuracies in predicted PFOS concentrations in some tissues over the first few days following drastic shifts in exposure.

Looking ahead, the DAM shows promise for expansion to other PFAS compounds, such as PFHxS, and livestock species, such as swine or broiler chickens. Future research should focus on validating the model across diverse exposure scenarios and developing a multi-compartment version for dairy cattle as protein binding, absorption, elimination rate, enterohepatic circulation, and other physiological data specific to dairy cattle becomes available. Additionally, efforts to integrate the DAM with models of environmental PFAS fate and transport could provide a more holistic approach to assessing PFAS risks in agricultural systems.

In conclusion, while acknowledging its limitations, the DAM stands as a significant contribution to the field, offering a flexible and practical tool for understanding and managing PFAS exposure in livestock. Its novelty lies in its balance of simplicity and comprehensiveness, making it a valuable tool for regulatory decision-making and risk assessment. As the field of PFAS research continues to evolve, the DAM provides a foundation for further refinement and expansion, potentially leading to more comprehensive and accurate risk assessment models in the future. The authors emphasize the need for actual exposure scenarios with PFAS concentrations in dietary sources, highlighting an area for future improvement. Ultimately, the DAM represents an important step forward in our ability to predict and manage PFAS contamination in livestock, contributing to broader efforts in ensuring food safety.

## Figures and Tables

**Table 1:** Weighting of half-life data for PFOS. The total weight is calculated by Exposure × Type × Tissue × Duration/Divisor × 100. Total Weights were used to select the number of samples for the overall distribution in Figure 1.

Study	Tissue	Half Life	SD	Exposure	Type	Tissue	Duration	Divisor	Total Weight
[52]	Body	114.2	22.84	1	3	3	1	1	9
[46]	Steer Plasma	120	4.1	1	3	1	3	3	3
[46]	Heifer Plasma	106	23.1	1	3	1	3	3	3
[46]	Grouped Muscle	165	33	1	3	3	3	3	9
[47]	Serum	74.1	13.4	3	1	1	3	2	4.5
[47]	Muscle	77	15.4	3	1	3	3	2	13.5

**Table 2:** Daily accumulation model (DAM) parameters for PFOS in beef muscle.

Parameter	Unit	Value	SD
Half-life	d	108.9	40.5
Muscle:Body PFOS		0.231	0.090
Muscle:BW		0.361	0.117
Muscle:Serum Fraction		0.080	0.016
Birth Weight	kg	35.450	3.270
BW’	kg	0.5584 + 2×0.001787*d* − 3×1.549×10^−6^*d*^2^ − 4×1.332×10^−9^*d*^3^	×0.35
BW > 540 days	kg	*BW* + 0.32	0.112
Cow DMI	kg	−1×10^−4^*d*^2^ + 0.0196*d* + 11.697	×0.062
Milk Prod (Intake)	kg	−0.0000000257*d*^4^ + 0.0000163*d*^3^ − 0.003686*d*^2^ + 0.3024*d* + 1.445	×0.355
Soil Intake	kg	0.0243 × *DMI_F_*	0.005 × DMI
Water Intake (Lactating cows)	L	56.47	8.16
Milk Transfer Factor	d/kg	0.0213	
DMI < 66 days	kg	0	
DMI > 66 days	kg	5.4702 × ln(*d*) − 22.889	*BW* × 0.0013
Water Intake (Nursing Calves)	L/day	4 × *DMI_F_*	
Water Intake (Finishers)	L/day	40.84	12.87

## Data Availability

The original contributions presented in this study are included in the article/Supplementary Materials. Further inquiries can be directed to the corresponding author.

## Supplementary Materials

The following supporting information can be downloaded at: https://www.mdpi.com/article/10.3390/toxics13080649/s1.
